# Molecular Mechanism of HINTW (HINT Gene in W-Chromosome)-Mediated UBE2I (Ubiquitin Conjugating Enzyme E2 I) Interaction in Female Differentiation of Chicken Embryos

**DOI:** 10.3390/genes16040366

**Published:** 2025-03-22

**Authors:** Changhua Sun, Jiuzhou Song, Malik Ahsan Ali, Hongyan Sun, Yingjie Niu, Qisheng Zuo, Wei Han, Bichun Li, Kai Jin

**Affiliations:** 1 College of Biological and Chemical Engineering, Yangzhou Polytechnic College, Yangzhou 225009, China; 101762@yzpc.edu.cn; 2 Joint International Research Laboratory of Agriculture and Agri-Product Safety of Ministry of Education of China, Yangzhou University, Yangzhou 225009, China; malik364ahsan@hotmail.com (M.A.A.); hongyans2392@163.com (H.S.); niuyj@yzu.edu.cn (Y.N.); 006664@yzu.edu.cn (Q.Z.); yubcli@yzu.edu.cn (B.L.); 3 Key Laboratory of Animal Breeding Reproduction and Molecular Design for Jiangsu Province, College of Animal Science and Technology, Yangzhou University, Yangzhou 225009, China; 4 Institutes of Agricultural Science and Technology Development, Yangzhou University, Yangzhou 225009, China; 5 Department of Animal & Avian Sciences, University of Maryland, College Park, MD 20742, USA; songj88@umd.edu; 6 College of Bioscience and Biotechnology, Yangzhou University, Yangzhou 225009, China; 7 Poultry Institute, Jiangsu Institute of Poultry Sciences/Chinese Academy of Agricultural Sciences, Yangzhou 225003, China; hanwei830@163.com; 8 College of Biotechnology, Jiangsu University of Science and Technology, Zhenjiang 212100, China

**Keywords:** HINTW, UBE2I, female differentiation, chicken embryo, pull-down assay

## Abstract

Background/Objective: It has been established that HINTW plays a pivotal role in the female differentiation of chickens; nevertheless, the underlying molecular mechanism remains to be fully elucidated. Method: To investigate the role of HINTW in avian sex determination, a prokaryotic expression vector containing its key structural domain was constructed, and its in vitro expression was achieved. Pull-down assays were performed to capture interacting proteins from male and female gonadal tissues, followed by a silver staining analysis to compare interaction profiles between ovaries and testes. Mass spectrometry was utilized to identify differentially bound proteins. Additionally, functional characterization and co-immunoprecipitation (Co-IP) assays were conducted to validate the interaction between HINTW and its candidate binding partner. Result: A total of 1590 differentially bound proteins were identified between ovarian and testicular tissues. Functional analysis and Co-IP assays confirmed a specific interaction between HINTW and UBE2I in the ovary, suggesting that HINTW may facilitate female differentiation in chicken embryos through its interaction with UBE2I. Conclusions: This study provides novel insights into the molecular mechanisms underlying HINTW-mediated female differentiation in chickens and contributes to a deeper understanding of avian sex determination.

## 1. Introduction

**Figure 1:**
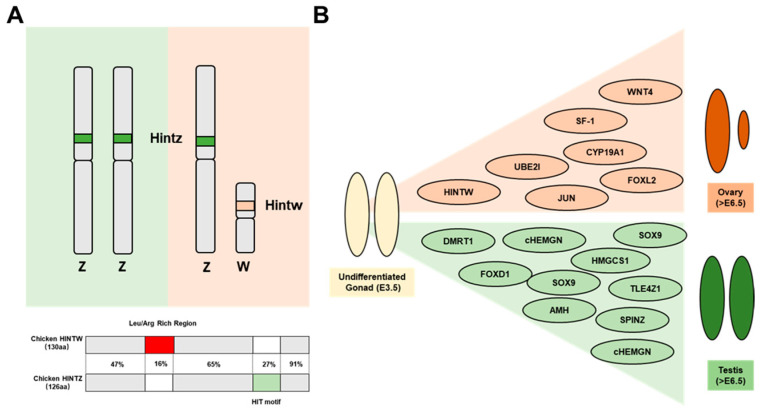
The structure and function of the HINTW in chicken sexual development: (**A**) Schematic representation of the deduced chicken HINTW and HINTZ proteins, highlighting regions of homology. The HIT motif in HINTZ is shaded in green, while the leucine- and arginine-rich region in HINTW is highlighted in red. (**B**) Gene expression during gonadal sexual development. Genes involved in male differentiation are indicated by green dots, while those associated with female differentiation are marked with red dots.

HINTW (histidine triad nucleotide-binding protein, W-linked) is located on the W chromosome of chickens and was initially identified independently by two research groups, named Wpkci (W-Linked Protein Kinase C Inhibitor) [1] and Asw (Avian Sex Related, W-link) [2]. It has been established that this gene encodes a derivative of histidine triad nucleotide-binding protein (HINT) rather than a protein kinase C inhibitor (PKCI), thus favoring HINTW as the more appropriate and widely accepted nomenclature [3]. Both HINTW and Hintz are homologous to mammalian HINT gene and belong to the HIT (histidine triad) protein superfamily [1,2,4]. In contrast to Hintz on the Z chromosome, HINTW lacks the histidine triad motif, rendering it incapable of catalyzing AMP-lysine conjugation (Figure 1A).

During chicken embryonic development, HINTW is specifically and highly expressed in the gonads and urogenital tract of female embryos, particularly before the initiation of gonadal differentiation [2,5]. This spatiotemporal expression pattern suggests that HINTW may play a crucial role in female sex differentiation and ovarian development (Figure 1B) [6,7,8,9,10]. Notably, the aromatase inhibitor treatment of ZW individuals results in the induction of male-like phenotypes, yet HINTW expression remains elevated [11], suggesting its involvement in broader sex regulatory networks extending beyond female sex determination. However, the precise molecular mechanisms through which HINTW contributes to avian sex determination remain to be elucidated and require further investigation [7,12,13,14].

In previous studies, HINTW was confirmed to participate in the early sex determination process in chicken embryos [5,7]. In order to further elucidate its molecular mechanism, a His-tagged HINTW vector was constructed, and pull-down assays were employed to detect protein-binding proteins in male and female chicken gonads. These interactions are subsequently validated through co-immunoprecipitation (Co-IP) assays to explore the functional role of HINTW in chicken embryonic sex determination. The findings not only provide insights into the molecular mechanism of HINTW but also offer new perspectives on the evolutionary conservation and diversity of avian sex determination mechanisms.

## 2. Materials and Methods

### 2.1. Ethics Statement

All the procedures involving the care and use of animals were conducted in accordance with the guidelines established by the U.S. National Institute of Health (NIH Pub. No. 85-23, revised 1996) and were approved by the Laboratory Animal Management and Experimental Animal Ethics Committee of Yangzhou University. The fertilized eggs of *Gallus gallus domesticus* were procured from the Poultry Research Institute of the Chinese Academy of Agricultural Sciences and subsequently hatched in an environment maintained at 37 °C with 70% humidity.

### 2.2. Expression and Detection of His-HINTW Protein

The constructed His-HINTW vector was used for protein expression. The bacterial culture was inoculated into LB medium and agitated overnight at 37 °C and 200 rpm. Thereafter, the temperature was reduced to the induction temperature, and IPTG was added to induce protein expression. After 3 h of induction, the culture was centrifuged at 8000× *g* at 4 °C for 3 min. The supernatant was discarded, and the pellet was resuspended in buffer. Lysozyme was then added, and the mixture was incubated on ice for 30 min. The sample was then sonicated and centrifuged at 16,000× *g* at 4 °C for 30 min. A small sample was taken for SDS-PAGE analysis, and the remaining sample was used for subsequent experiments.

### 2.3. Protein Purification and Concentration

Initially, the HIS agarose gel was loaded into a chromatography column, which was subsequently washed with a volume of buffer 10 times the capacity of the column bed. Thereafter, the sample supernatant was introduced into the column at a flow rate of 0.5 mL/min. The column was washed with a volume of buffer 10 times the capacity of the column bed at a flow rate of 1 mL/min in order to remove any impurities. Finally, the protein was eluted with 10 times the column bed volume of GSH buffer at a flow rate of 1 mL/min. The eluted fractions were collected and analyzed by SDS-PAGE to check the protein components, and the protein was concentrated.

### 2.4. His Pull-Down Assay

As the His tag is capable of binding to a Ni column, proteins that interact with His-tagged fusion proteins will be adsorbed onto the Ni column during the pull-down assay. This allows for their subsequent separation and identification. The experimental steps are briefly described below: First, the Ni column was washed with pre-cooled PBS (4 °C), mixed, and centrifuged at 2500× *g* for 3 min, with the supernatant discarded. This step was repeated once, and the Ni column was then resuspended in pre-cooled PBS to a 50% slurry, mixed and stored on ice for later use. According to the experimental design, the recombinant His-HINTW protein was mixed with the target tissue samples for incubation. PBST (PBS containing Tween-20) was used to wash the Ni column, followed by centrifugation at 2500× *g* for 3 min at 4 °C. The supernatant was discarded, and the washing was repeated three times. The corresponding tissue samples were then added and incubated overnight at 4 °C. After incubation, the sample was centrifuged at 2500× *g* for 3 min at 4 °C, and the supernatant was discarded. The washing step was repeated three times with PBST. Loading protein buffer was added after thoroughly mixing the pellet with RIPA protein lysis buffer. The samples were heated for 10 min, centrifuged at 12,000× *g* for 5 min, and the supernatant was collected. The samples were analyzed by SDS-PAGE and Western blot. The ECL detection kit (Pierce ECL Western blotting Substrate) was used for visualization, and images were captured using a Bio-Rad ChemiDoc™ Imaging System (Bio-Rad Laboratories, Hercules, CA, USA). Images were analyzed using Image Lab 6.0 software. Finally, the differentially bound protein samples from the pull-down assay were sent to Jinkairui Biological Engineering Co., Ltd. (Wuhan, China) for mass spectrometry analysis to identify potential interacting proteins. The peptide over FDR < 0.01 was the highly expressed protein.

### 2.5. Mass Spectrometry (MS) Data Acquisition and Analysis

Lyophilized fractions were obtained through in-gel enzymatic digestion and reconstituted in 0.1% formic acid (FA). The reconstituted samples were centrifuged at 14,000× *g* for 20 min, and the supernatant was collected for mass spectrometry analysis. The raw mass spectrometry (MS) data files were processed using Proteome Discoverer 2.4 for qualitative and quantitative analysis, with *Uniprot_Gallus gallus* (2024) as the reference database. Peptide-spectrum matches with an ion score above the identification threshold (FDR < 0.01) were classified as Identified-Spectra. Non-redundant peptide sequences derived from these spectra were defined as Identified-Peptides, while the corresponding proteins containing at least one unique identified peptide were designated as Identified-Proteins. Further functional enrichment analysis was performed using the STRING database.

### 2.6. Co-Immunoprecipitation (Co-IP)

Following the construction of the HINTW expression vectors, fusion expression plasmids were constructed using the pcDNA3.1 vector. The HINTW and Hintz sequences were separately inserted into the His and Flag tags of the vectors and then transformed and cloned. After colony picking and sequencing, the plasmids were named His-HINTW and Flag-HINTZ. Following transfecting of His-HINTW and Flag-HINTZ into DF-1 cells, total protein was collected for Western blot analysis (as mentioned above) to detect fusion protein expression using Flag and His tag antibodies. The immunoprecipitation method was performed according to the instructions of the Thermo Scientific™ Pierce^®^ Immunoprecipitation (Co-IP) Kit (Thermo Fisher Scientific, Waltham, MA, USA), the detailed procedure as previous study [15].

## 3. Results

### 3.1. The Construction and Identification of the pET-SUMO-HINTW

**Figure 2:**
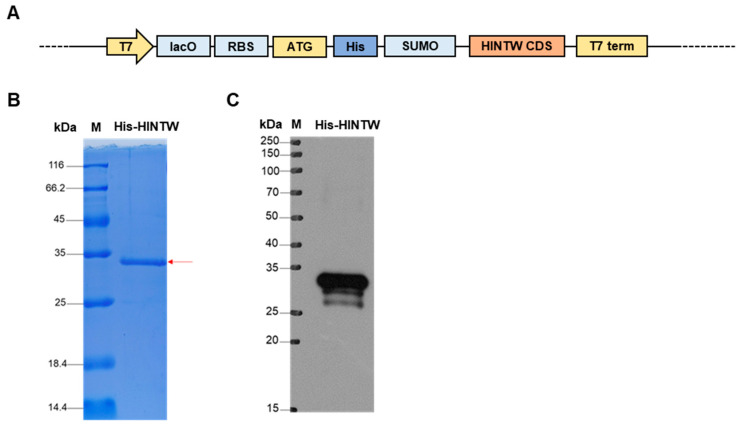
The construction and identification of the pET-SUMO-HINTW: (**A**) The map of pET-SUMO-HINTW plasmid. (**B**) Coomassie brilliant blue staining to detect the expression of His-HINTW fusion protein. The red arrow points the His-HINTW protein. (**C**) Western blot detects the size of His-HINTW fusion protein.

To further investigate the mechanism by which HINTW functions as a protein, in the absence of a specific HINTW antibody, the CDS region of HINTW (130 amino acids, 13.9 kDa) [16] was cloned into the pET-SUMO vector containing His and SUMO tags (~12 kDa), resulting in a His-HINTW fusion protein with an expected molecular weight of approximately 25.9 kDa (Figure 2A). The constructed pET-SUMO-HINTW prokaryotic expression vector was used to induce the expression of the His-tagged fusion protein. After induction, the bacterial lysate was subjected to precipitation and supernatant separation, followed by Coomassie Brilliant Blue staining of the processed protein samples. The results showed a protein band of the expected size, indicating successful fusion protein expression (Figure 2B). Subsequent Western blot analysis further confirmed the expression of the His-HINTW fusion protein with the expected molecular weight, demonstrating that the pET-SUMO-HINTW expression vector was successfully constructed and capable of expressing the target protein (Figure 2C).

### 3.2. The Identification of the HINTW Interaction Protein via Pull-Down

**Figure 3:**
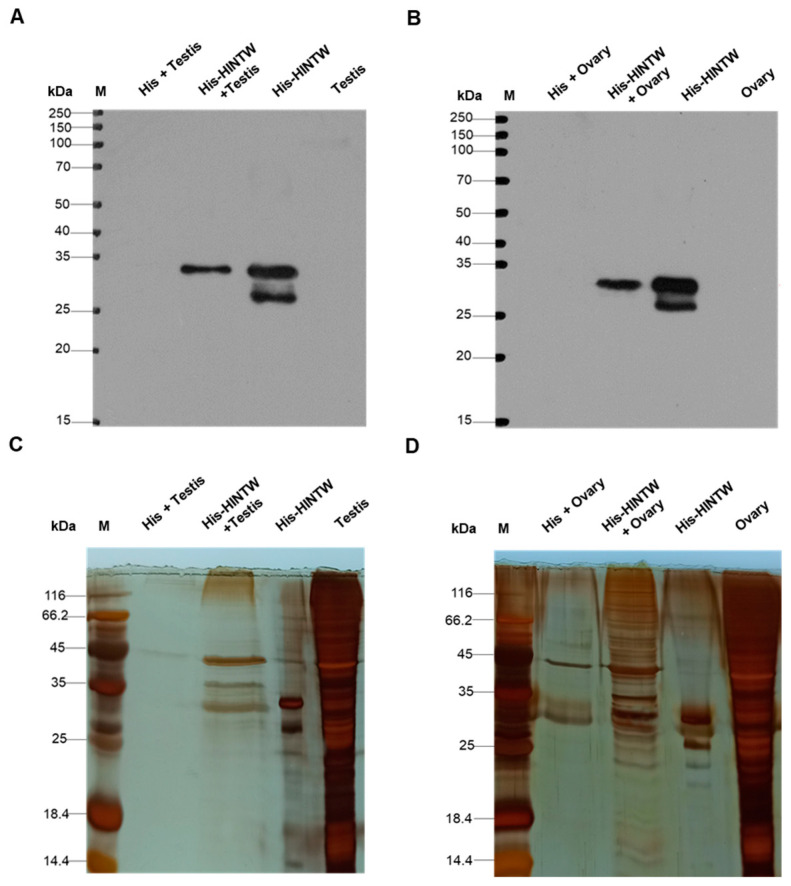
The identification of the HINTW interaction protein via pull-down: (**A**) Western blot detects the interaction protein between His-HINTW fusion protein after pull-down in testis tissue (M: protein marker; His+Testis group: His-beads + chicken testis total protein; His-HINTW+Testis group: His-HINTW protein + beads + chicken testis total protein; His-HINTW group: His-HINTW protein; and Testis group: total protein of chicken testis tissue). (**B**) Western blot detects the interaction protein between His-HINTW fusion protein after pull-down in ovarian tissue (M: protein marker; His + Ovary group: His-beads + chicken ovarian total protein; His-HINTW + Ovary group: His-HINTW protein + beads + chicken ovarian total protein; His-HINTW group: His-HINTW protein; and Ovary group: total protein of chicken ovarian tissue). (**C**) Silver stain detects the interaction protein between His-HINTW fusion protein after pull-down in testis tissue (M: protein marker; His+Testis group: His-beads + chicken testis total protein; His-HINTW + Testis group: His-HINTW protein + beads + chicken testis total protein; His-HINTW group: His-HINTW protein; and Testis group: total protein of chicken testis tissue). (**D**) Silver stain detects the interaction protein between His-HINTW fusion protein after Pull-down in ovarian tissue (M: protein marker; His + Ovary group: His-beads + chicken ovarian total protein; His-HINTW + Ovary group: His-HINTW protein + beads + chicken ovarian total protein; His-HINTW group: His-HINTW protein; and Ovary group: total protein of chicken ovarian tissue).

The pull-down assays were performed using the His protein and His-HINTW fusion protein in the testes and ovarian tissues of bisexual chicken embryos, respectively, and the pulled-down interacting proteins were detected. Western blot results showed that His-tagged interacting proteins could be detected in both the ovarian and testicular experimental groups, indicating that the His antibody successfully pulled down the proteins interacting with the His-HINTW fusion protein (Figure 3A,B). Further silver staining results also revealed that there were numerous visually distinguishable differential proteins between the control and experimental groups among the His-tagged interacting proteins, suggesting that further mass spectrometry analysis could be performed (Figure 3C,D).

### 3.3. The Analysis of HINTW Interacting Proteins

**Figure 4:**
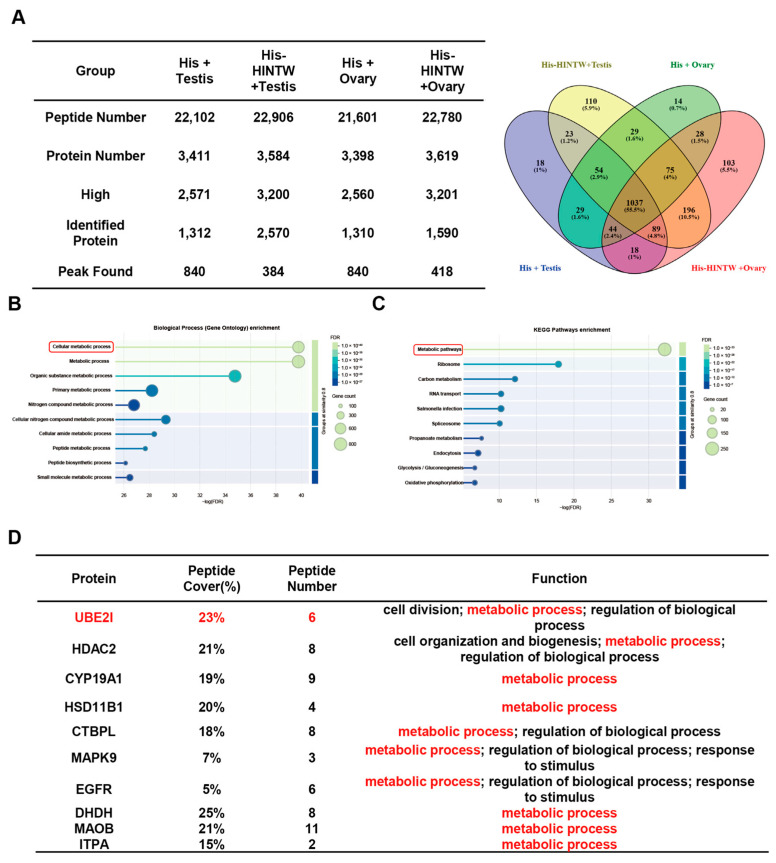
Mass spectrometry for HINTW HIS pull-down protein: (**A**) The number of the peptide and identified protein. (**B**) The biological process enrichment of identified protein. Note: the red box means the cellular metabolic process. (**C**) The KEGG pathway enrichment of identified protein. Note: the red box means the metabolic pathway. Note: the red box means the metabolic pathway. (**D**) The peptide cover rate of partially identified protein. Note: the red character means the metabolic process.

Mass spectrometry analysis was performed to compare and detect the interacting proteins obtained from the pull-down assay. Different numbers of peptides were identified in various experimental groups, and further identification revealed specific proteins, indicating that the HINTW protein can perform its function in the ovary by interacting with different proteins (Figure 4A, Tables S1 and S2). Further functional analysis of HINTW-binding proteins in the ovary showed that the pathways and expression patterns of these differential proteins (UBE2I, HDAC2, CYP19A1, HSD11B1, CTBPL, MAPK9, EGFR, DHDH, MAOB and ITPA) were significantly enriched in metabolic pathways (Figure 4B,C, Tables S3 and S4). Additionally, the analysis of proteins within the metabolic pathways revealed that the UBE2I protein interacts with the HINTW protein in the ovary, suggesting that HINTW is likely to participate in the female sex determination process in chick embryos by interacting with UBE2I (Figure 4D).

### 3.4. Indirect Immunofluorescence Staining for PGCLCs

**Figure 5:**
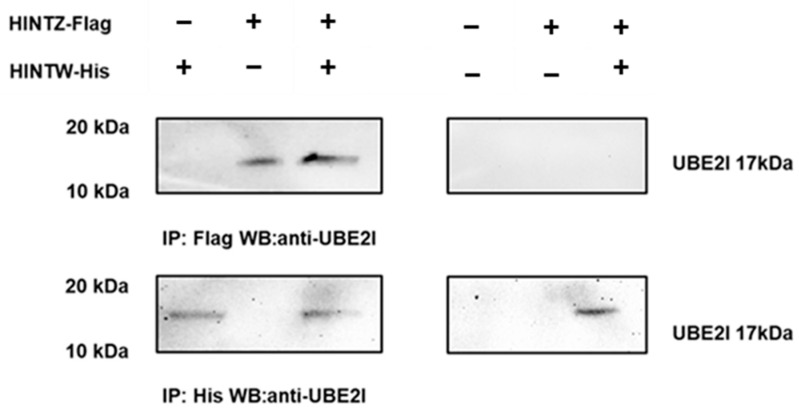
Immunoprecipitation to detect the interaction between His-HINTW fusion protein and UBE2I in DF-1 cells.

To confirm whether there is a true interaction between HINTW and UBE2I, we transfected His-HINTW and Flag-HINTZ into DF-1 cells and in both male and female CEF cells in different group combinations. After 48 h of transfection, proteins were extracted, and immunoprecipitation was performed using His and Flag antibodies. The complexes were detected by Western blot. The results showed that HINTW could bind to UBE2I in both DF-1, indicating that HINTW can interact with UBE2I (Figure 5).

## 4. Discussion

To further investigate the specific regulatory mechanism of HINTW in the process of sex differentiation, this study used pull-down technology combined with mass spectrometry to screen for proteins interacting with the HINTW protein in the testes and ovaries. The results revealed that the selected interacting proteins included important genes such as UBE2I [17,18], CYP19A1 [19,20,21], MAPK9 [22] and HDAC2 [23,24]. These proteins have been confirmed to be involved in the sex differentiation process and are widely distributed in developmental and hormone synthesis-related pathways. Based on these findings, their specific functional mechanisms have been further investigated.

In this experiment, we first constructed a His-HINTW expression vector with a His tag for prokaryotic expression, performing large-scale expression, purification, and identification in vitro. Meanwhile, we used a blank control for mass spectrometry analysis and screening of the differentially binding proteins. The mass spectrometry results identified a large number of differentially bound proteins, suggesting that HINTW exerts its functions by interacting with various proteins. Further studies showed that HINTW can bind to UBE2I in the ovary, and Co-IP experiments further confirmed the accuracy of the mass spectrometry results. This suggests that HINTW functions by binding to UBE2I and that UBE2I also plays an important role in chicken embryonic sex determination [25].

UBE2I is located on chicken chromosome 14 and may be involved in physiological functions such as cell growth, differentiation, and apoptosis by affecting the localization of target proteins and protein–protein interactions [26,27,28]. Studies have shown that UBE2I expression tends to decrease and then increase during mouse oocyte growth [29]. Previous research confirmed that UBE2I is highly expressed in female germ cells and that its expression level in female gonads is significantly higher than in other tissues, suggesting that UBE2I is involved in female sex differentiation [12]. In this experiment, His pull-down and mass spectrometry analysis successfully identified the interaction between the UBE2I of HINTW proteins. Co-IP also confirmed that HINTW can bind to UBE2I in female cells. This indicates that HINTW specifically interacts with UBE2I in the female ovary and is involved in early female sex determination in chickens.

## 5. Conclusions

Taken together, these studies demonstrate that HINTW directly interacts with UBE2I, a key gene involved in female development within the chicken ovary. While these findings provide novel insights into the molecular mechanisms of HINTW-mediated female differentiation in chickens and contribute to a deeper understanding of avian sex determination, and the precise underlying mechanisms require further investigation.

## Data Availability

The data that support the findings of this study are available from the corresponding author upon reasonable request.

## Supplementary Materials

The following supporting information can be downloaded at https://www.mdpi.com/article/10.3390/genes16040366/s1, Figure S1: The total ions chromatogram (TIC) of Pull-down Sample; Table S1: The Identified Peptide groups of Pull-down Sample; Table S2: The Identified Protein groups of Pull-down Sample; Table S3: The Enrichment of Biological Process; and Table S4: The Enrichment of KEGG pathway.
