# Supplementary figures and images for: Molecular Mechanism of HINTW (HINT Gene in W-Chromosome)-Mediated UBE2I (Ubiquitin Conjugating Enzyme E2 I) Interaction in Female Differentiation of Chicken Embryos
Source: Genes (Basel). 2025 Mar 22;16(4):366. doi: 10.3390/genes16040366 (PMC12026566; doi:10.3390/genes16040366)

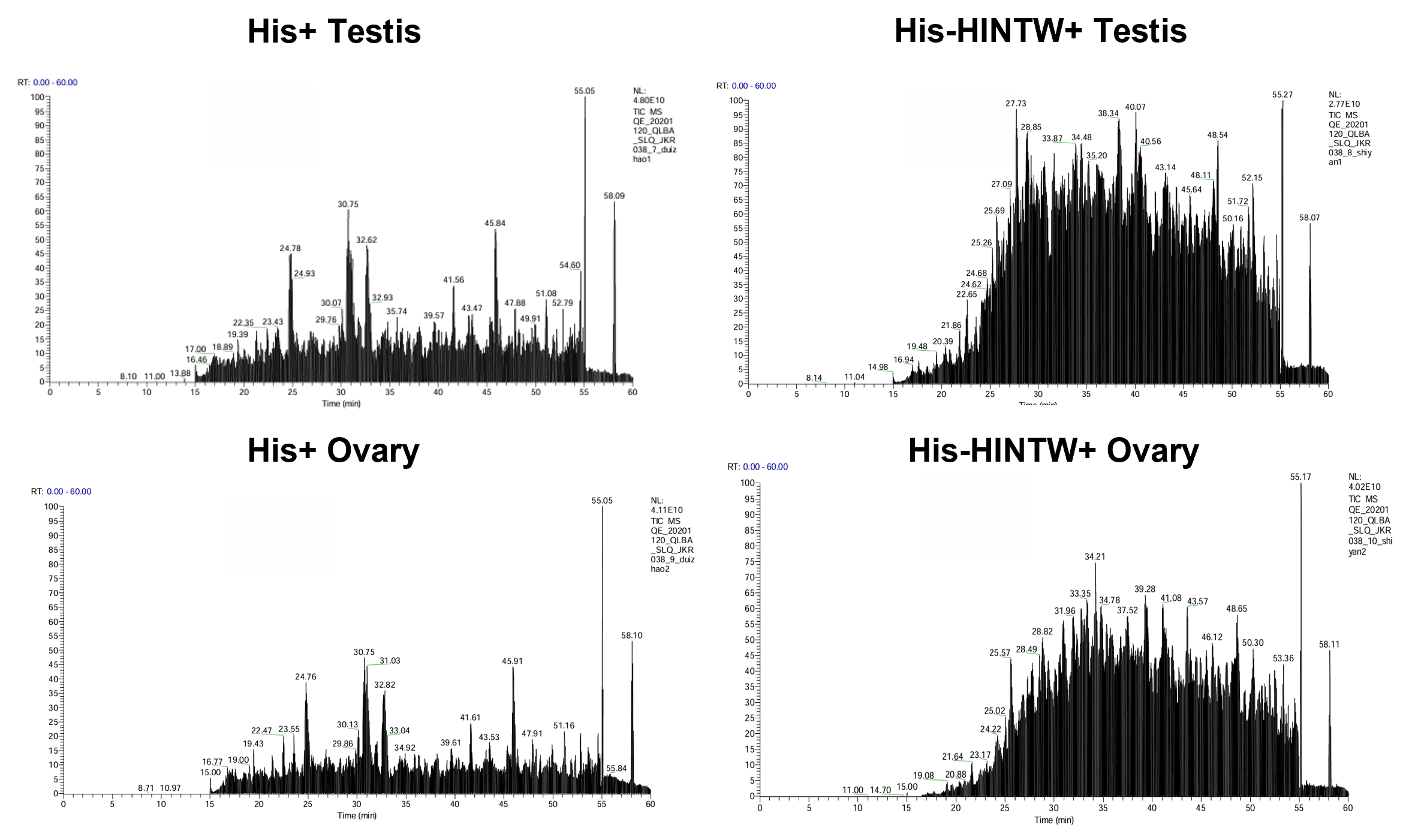

Supplement: Supplementary file 1 [file genes-16-00366-s001.zip › Figure S1.png]
